# Graph Learning for Cortical Parcellation from Tensor Decompositions of Resting-State fMRI

**DOI:** 10.1101/2024.01.05.574423

**Published:** 2024-01-17

**Authors:** Yijun Liu, Jian Li, Jessica L. Wisnowski, Richard M. Leahy

**Affiliations:** a Ming Hsieh Department of Electrical and Computer Engineering, University of Southern California, Los Angeles, CA, USA.; b Athinoula A. Martinos Center for Biomedical Imaging, Massachusetts General Hospital and Harvard Medical School, Charlestown, MA, USA.; c Center for Neurotechnology and Neurorecovery, Department of Neurology, Massachusetts General Hospital and Harvard Medical School, Boston, MA, USA.; d Radiology and Pediatrics, Division of Neonatology, Children’s Hospital Los Angeles, Los Angeles, CA, USA.; e Keck School of Medicine, University of Southern California, Los Angeles, CA, USA.

**Keywords:** Cortical parcellation, Resting-state fMRI, Temporal synchronization, Tensor decomposition, Graph representation learning

## Abstract

Cortical parcellation has long been a cornerstone in the field of neuroscience, enabling the cerebral cortex to be partitioned into distinct, non-overlapping regions that facilitate the interpretation and comparison of complex neuroscientific data. In recent years, these parcellations have frequently been based on the use of resting-state fMRI (rsfMRI) data. In parallel, methods such as independent components analysis have long been used to identify large-scale functional networks with significant spatial overlap between networks. Despite the fact that both forms of decomposition make use of the same spontaneous brain activity measured with rsfMRI, a gap persists in establishing a clear relationship between disjoint cortical parcellations and brain-wide networks. To address this, we introduce a novel parcellation framework that integrates NASCAR, a three-dimensional tensor decomposition method that identifies a series of functional brain networks, with state-of-the-art graph representation learning to produce cortical parcellations that represent near-homogeneous functional regions that are consistent with these brain networks. Further, through the use of the tensor decomposition, we avoid the limitations of traditional approaches that assume statistical independence or orthogonality in defining the underlying networks. Our findings demonstrate that these parcellations are comparable or superior to established atlases in terms of homogeneity of the functional connectivity across parcels, task contrast alignment, and architectonic map alignment. Our methodological pipeline is highly automated, allowing for rapid adaptation to new datasets and the generation of custom parcellations in just minutes, a significant advancement over methods that require extensive manual input. We describe this integrated approach, which we refer to as *Untamed*, as a tool for use in the fields of cognitive and clinical neuroscientific research. Parcellations created from the Human Connectome Project dataset using *Untamed*, along with the code to generate atlases with custom parcel numbers, are publicly available at https://untamed-atlas.github.io.

## 1 Introduction

Cortical parcellation, which partitions the cerebral cortex into discrete, non-overlapping regions, is pivotal for the interpretation and comparison of neuroscientific findings. It simplifies the computational demand posed by high-dimensional neuroimaging data and is instrumental in elucidating cerebral organization. This endeavor has captivated the neuroscience community’s interest for many years as evidenced by extensive research in this domain (Brodmann, 1909; Tzourio-Mazoyer et al., 2002; Yeo et al., 2011; Glasser et al., 2016; Schaefer et al., 2018; Eickhoff et al., 2018; Han et al., 2020; Joshi et al., 2022).

Resting-state functional magnetic resonance imaging (rsfMRI) has become a cornerstone technique for non-invasively measuring spontaneous in vivo brain activity. By recording blood-oxygen-level-dependent (BOLD) signals without task stimuli, rsfMRI has proven instrumental in deciphering the human brain’s intrinsic functional brain networks (Biswal et al., 1995; Beckmann et al., 2005; Allen et al., 2014). It is also a commonly used tool to derive both group (Yeo et al., 2011; Craddock et al., 2012; Shen et al., 2013; Gordon et al., 2016; Schaefer et al., 2018; Yan et al., 2023) and individual (Chong et al., 2017; Kong et al., 2019) parcellations. However, despite the extensive research on functional brain networks and brain parcellation, there remains a notable gap in explicitly linking these two inherently interconnected topics. One recent study (Fan et al., 2021) attempted to bridge the connection between the two by deriving parcellation from independent component analysis (ICA) of dynamic functional connectivity. Nevertheless, due to the nature of signal decompositions using two-way (space × time) tensor, or equivalently matrix, representations with ICA, a statistical spatial or temporal independence constraint is imposed to perform the network decomposition (Calhoun et al., 2001; Varoquaux et al., 2010; Smith et al., 2014). This condition may not be inherently consistent with real interacting functional brain networks.

In order to bridge the connection between functional brain networks and cortical parcellations without imposing implausible constraints, such as statistical independence or orthogonality (Smith et al., 2014), our approach integrates NASCAR (Li et al., 2021, 2023) with a novel graph-learning method. NASCAR computes a three-way tensor decomposition of temporally aligned rsfMRI data across subjects (Joshi et al., 2018; Akrami et al., 2019), facilitating the identification of functional networks that may more authentically represent brain activity patterns by avoiding the need to impose spatial and/or temporal independence between networks. The networks identified by NASCAR have been shown to be, to a large degree, consistent with those found using ICA but with subtle differences that are consistent with other findings in the literature, such as subsystems of the default-mode network found using seed-based clustering and Bayesian methods (Andrews-Hanna et al., 2010; Andrews‐Hanna et al., 2014; Buckner and DiNicola, 2019; Harrison et al., 2020). We also showed that the networks found using NASCAR can be used to better classify subjects with neurological disorders (Li et al., 2023). Based on the brain networks identified by NASCAR, we then construct a graph and apply state-of-the-art graph representation learning method to produce cortical parcellations.

Recent advances in graph representation learning have shown superior performance in a variety of applications, including social networks, natural language processing, and biomedicine. However, these emerging graph learning methods have not yet been fully exploited in brain parcellation methodology. A few studies that have applied graph representation learning to brain parcellation lack comprehensive comparisons with other parcellation schemes (e.g., Gopinath et al., 2019; He et al., 2020; Zhang and Wang, 2019). Therefore, it is difficult to gauge their potential advantage with respect to neuroscientific studies over other more widely used approaches. Here, we use the state-of-the-art network embedding method “Network embedding as Matrix Factorization” (NetMF) (Qiu et al., 2018). We also compared our parcellation series to an extensive set of widely-used parcellations in terms of commonly-used metrics such as Resting State Function Connectivity (RSFC) homogeneity, task contrast alignment, and architectonic map alignment. Our findings indicate that our parcellations either outperform or are on par with some of the most widely used atlases in neuroscience, including the Schaefer atlases (Schaefer et al., 2018). We also discuss the rather surprising observation that, despite the marked differences in the boundary locations of our parcellations and those of the Schafer atlases, for example, they exhibit remarkably similar performance in terms of the metrics listed above.

Our methodological pipeline consists of temporal synchronization or alignment using BrainSync (Joshi et al., 2018; Akrami et al., 2019), brain network tensor decomposition using NASCAR (Li et al., 2023), and graph representation learning. The pipeline is largely automated, in contrast with several alternative parcellation methods (e.g., Glasser et al., 2016; Joshi et al., 2022) that require expert manual input. Users can apply our framework to new datasets with any desired number of parcels. Once brain networks are computed using NASCAR, it takes only a few minutes on a modern workstation to generate an atlas with the desired number of parcels. Since we leverage the **u**nconstrained spatial activation maps from **N**ASCAR with **t**emporal **a**lignment plus node e**m**b**ed**ding, we named our cortical parcellations *Untamed*.

## 2 Material and methods

### 2.1 Overview

**Fig. 1.**
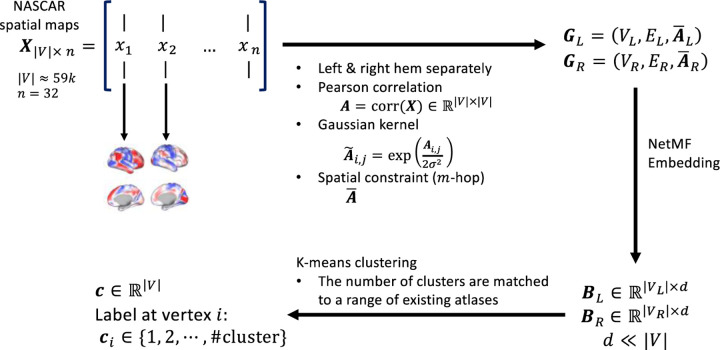
Pipeline for generating *Untamed* parcellations from HCP rsfMRI data. The input to the pipeline are the spatial activation maps from BrainSync and NASCAR applied to rsfMRI data of 500 subjects in HCP dataset as described in (Li et al., 2023).

We derived cortical parcellations using rsfMRI data from 500 subjects from the Human Connectome Project (HCP) dataset (Van Essen et al., 2012; Glasser et al., 2013). The procedure includes a temporal alignment using BrainSync (Akrami et al., 2019; Joshi et al., 2018), a 3-way tensor decomposition using NASCAR (Li et al., 2021, 2023), graph construction from spatial maps followed by graph node embedding using NetMF (Qiu et al., 2018), and finally *k*-means clustering to obtain the parcellations. The overall pipeline is depicted in Fig. 1. The rsfMRI data from an independent set of 500 subjects from the HCP were used for evaluation. In addition to the HCP dataset, we also evaluated the parcellation performance on the Yale rsfMRI (Lee et al., 2022) and the multi-domain task battery (MDTB) (King et al., 2019; Zhi et al., 2022) datasets. In this way, we avoid potential bias in the evaluation of Untamed when comparing to other parcellations that may arise from using data collected using the HCP protocol for both construction and evaluation of parcellations.

### 2.2 Datasets

#### 2.2.1 The Human Connectome Project (HCP) dataset

We utilized the minimally preprocessed 3T resting-state fMRI (rsfMRI) data from 1000 subjects (466 males, 534 females, age between 22 and 35) from the Human Connectome Project (HCP) (Glasser et al., 2013; Van Essen et al., 2012). The rsfMRI data were acquired with *TR* = 0.72 s, *TE* = 33.1 ms and a 2 mm isotropic resolution and co-registered onto a common atlas in MNI space (Glasser et al., 2013). We used the scans acquired in the LR phase encoding direction. Each session ran 15 minutes with 1200 time points. The data was resampled onto the cortical surface extracted from each subject’s T1-weighted MRI and co-registered to a common surface (Glasser et al., 2013). No additional spatial smoothing was applied other than the 2 mm FWHM isotropic Gaussian smoothing in the minimal preprocessing pipeline (Glasser et al., 2013). We randomly split the 1000 HCP subjects into two equal-sized independent groups, each with 500 subjects. The rsfMRI data from the first group were employed to extract shared spatial maps and to create cortical parcellations. This group is referred to as the “training set” for brevity, though it should be noted that our method is entirely unsupervised without any labeled training. The second group is designated as the “test set”, which we used for the aseessment of parcellations. Aside from rsfMRI data, we also utilized the group-average task activation z-score maps from HCP (Barch et al., 2013) for evaluation. There are seven tasks spanning various domains from which we used the following unique contrasts (one for each term in brackets): working memory (body, face, place, tool), gambling (punish, reward), motor (left foot, left hand, right foot, right hand, tongue), language (math, story), social (random, tom), relational (match, relational), emotion (faces, shapes).

#### 2.2.2 Yale resting-state fMRI dataset

We used the Yale rsfMRI dataset (Lee et al., 2022) as an independent dataset for assessing RSFC homogeneity (Schaefer et al., 2018). The dataset comprises 27 subjects (11 males, 16 females, aged between 22 and 31). Each subject underwent two rsfMRI scans, each with *TR* = 1s, *TE* = 30ms, and a 2 mm isotropic resolution. The total duration of each session was 6 minutes 50 seconds, encompassing 410 frames. We preprocessed the data using fMRIPrep (Esteban et al., 2019), which sampled the fMRI data onto the fs_LR 32K surfaces compatible with the HCP dataset. Four subjects’ data were not successfully preprocessed by the fMRIPrep pipeline due to corrupted files and were consequently excluded from the evaluation. Following the recommendations from the curators of the dataset, we discarded the first 10 seconds of each scan and temporally concatenated the rsfMRI data of the two sessions for each subject.

#### 2.2.3 Multi-domain task battery (MDTB) dataset

We employed the multi-domain task battery (MDTB) dataset (King et al., 2019; Zhi et al., 2022) for evaluating the alignment between parcels in the atlases and areas of activation in the task activation maps. The MDTB dataset contains task fMRI for 24 healthy subjects (8 males, 16 females, mean age 23.8 years old) conducting 26 tasks, including motor, language, and social domains. The fMRI data were acquired on a 3T Siemens Prisma scanner with *TR* = 1s, 3 mm slice thickness, and 2.5 × 2.5 mm^2^ in-plane resolution. The contrast maps were derived using general linear modeling (GLM) based on the task designs and are available from the database, already resampled to the fs_LR 32K space.

### 2.3 Tensor-based identification of Brain Networks using NASCAR and BrainSync

The multisubject rsfMRI data are organized as a three-way tensor (space × time × subject). In order to identify a low-rank model via tensor decomposition, the data should be both temporally and spatially aligned. Spatial alignment is straightforward using standard inter-subject image registration methods, but the spatially-aligned time series for rsfMRI will still be independent across subjects. However, if they share a common network structure as reflected in their spatial correlation patterns, we can perform an orthogonal transform that will temporally align or synchronize the data across subjects. This alignment is accomplished using the BrainSync transform (Joshi et al., 2018). BrainSync computes an orthogonal transformation between fMRI recordings from a pair of subjects such that the sum of squared errors between their aligned time series is minimized. As a result, the time series in homologous brain locations will be highly correlated after the BrainSync transform is applied (and perfectly so if the two datasets have identical correlations). A multi-subject extension that minimizes squared error from all subjects to an automatically generated group template is described in (Akrami et al., 2019), which is the method used here to align all subjects that are used to generate the cortical parcellation.

After temporal synchronization, we formed a third-order tensor $𝒳\in {\mathbb{R}}^{\left|V\right|\times T\times S}$ from the spatially aligned and temporally synchronized rsfMRI data, where V is the set of cortical vertices with a cardinality $\left|V\right|\approx 59K$, T=1200 is the number of time points, S=500 is the number of subjects used to generate the atlas. We then applied NASCAR to decompose the group rsfMRI data into a set of brain networks common to all subjects using a Canonical Polyadic model (Kolda and Bader, 2009; Cichocki et al., 2015; Li et al., 2021, 2023)

(1) $$𝒳\approx \sum_{r=1}^{R}\lambda_{r}a_{r}\circ b_{r}\circ c_{r}$$

where each outer product $\lambda_{i}a_{i}\circ b_{i}\circ c_{i}$ represents a brain network: $a_{i}\in {\mathbb{R}}^{\left|V\right|}$ are the spatial network maps, $b_{i}\in {\mathbb{R}}^{T}$ their (synchronized) temporal dynamics, and $c_{i}\in {\mathbb{R}}^{S}$ the subject participation level for the *i*^th^ network; $\lambda_{i}$ represents the network magnitude, indicating the relative activity level with respect to other networks. In contrast to the commonly used blind source separation techniques such as PCA or ICA, NASCAR imposes neither orthogonality (as with PCA) (Smith et al., 2014) nor statistical independence (as with ICA) (Beckmann et al., 2005). The shared spatial network maps $\left\{a_{i}\right\}$ can be overlapped and correlated (Li et al., 2023). Based on our previous work that examined stability and reproducibility across data sets, we chose a rank *R*=35 (Li et al., 2023) representing 35 distinct networks. We performed NASCAR decomposition on the training and test set separately and obtained two sets of 35 spatial network maps. We then computed an optimal match between the two sets of maps using the Hungarian matching algorithm (Kuhn, 1955) and checked that each map in the first set had a correlation with its match from the second set > 0.5. One of the 35 maps failed to meet this criterion and was discarded. We also excluded two global maps that represent cardiac and respiratory factors rather than neural activity as discussed in (Li et al., 2023). After this selection procedure, 32 spatial networks maps were retained, forming a spatial feature matrix X with a dimension $\left|V\right|\approx 59K\times 32$, i.e., a 32-dimensional feature vector at each vertex. In other words, $X_{ij}$ represents the value at the *i*^th^ vertex in the *j*^th^ network map.

### 2.4 Graph construction from NASCAR spatial maps

We computed the Pearson correlation matrix $A=\text{corr}\left(X\right)\in {\mathbb{R}}^{\left|V\right|\times \left|V\right|}$ from the feature matrix X as a measure of similarity between feature vectors between each pair of nodes. We then computed an adjacency matrix, following (Ng et al., 2001), using the Gaussian kernel ${\tilde{A}}_{i,j}=\exp\left(\frac{A_{i,j}}{2\sigma^{2}}\right)$, σ=0.5. Our goal is to produce contiguous parcels, and as such, we are not interested in long-range correlations. Therefore, we generate a graph where connections are restricted using an *m*-hop spatial neighborhood constraint defined on the fs_LR 32K surface mesh. Specifically, we define ${\bar{A}}_{i,j}={\tilde{A}}_{i,j}$ for all (*i*, *j*) for which *i* is within *m* hops of *j* and zero otherwise.

In a related approach, (Craddock et al., 2012) used a 1 -hop neighborhood, retaining nearest neighbors only. However, we found that larger values *m* may produce better performance. We leave this as a hyperparameter in the algorithm, which can be tuned based on weighted average homogeneity (see the evaluation section). We experimented with values *m* between 1 and 10 with a step size of 1, and between 15 and 45 with a step size of 5. We found that *m* = 35 performed the best for parcel numbers smaller than 200 and *m* = 25 otherwise in the results presented below.

Using the above approach, we obtained one sparsely connected (*m*-hop) graph for each hemisphere: $G_{L}=\left(V_{L},{\bar{A}}_{L}\right)$ and $G_{R}=\left(V_{R},{\bar{A}}_{R}\right)$, where $V_{L}$, $V_{R}$ are the set of cortical vertices and ${\bar{A}}_{L}$, ${\bar{A}}_{R}$ are the *m*-hop filtered adjacency matrices of the graphs $G_{L}$, $G_{R}$, for the left and the right hemispheres, respectively.

### 2.5 Graph node embedding and clustering

We adopted the method outlined in NetMF (Qiu et al., 2018) to embed vertices in a lower dimensional space with more representative features that can be used for clustering. This approach (Qiu et al., 2018) established an equivalence between the widely-used DeepWalk algorithm (Perozzi et al., 2014) and matrix factorization techniques. Briefly, DeepWalk traversed vertices in the graph using a random walk. At each vertex during traversal, DeepWalk considers neighboring vertices as co-occurrent “positive” pairs as well as random node pairs outside the neighborhood as “negative samples.” It then applies a skip-gram model to train on the collected samples to derive the final embeddings. NetMF approximates DeepWalk by factorizing the following matrix (Qiu et al., 2018):

(2) $$M=\log\left(\max\left\{\frac{\sum_{i=1}^{\left|V\right|}\sum_{j=1}^{\left|V\right|}{\bar{A}}_{i,j}}{bT}\sum_{r=1}^{T}{\left(D^{-1}\bar{A}\right)}^{r}D^{-1},1\right\}\right)$$

where D is the degree matrix of the graph; log(∙) and max{∙, 1} are element-wise logarithmic and maximum operations, respectively; *b* denotes the number of negative samples; and *T* is the window length over which we consider nodes as co-occurrent positive pairs. We found that in practice the method is not sensitive to the choice of these two hyperparameters, which had little impact on the averaged training subjects’ RSFC homogeneity (see evaluation metrics section). We set *b* = 1, the default value in the original NetMF paper, and *T* = 7 as the value that maximized the homogeneity of average RSFC over the training subjects. We computed M from ${\bar{A}}_{L}$ and ${\bar{A}}_{R}$ separately. We used singular value decomposition (SVD) to obtain the final embeddings (Qiu et al., 2018). Specifically, let $M=U_{d}\Sigma_{d}V_{d}^{⊤}$, where $U_{d}$ and ${\text{Σ}}_{d}$ being the *d* largest singular values and their associated left singular vectors. The final embeddings were computed as $B=U_{d}\sqrt{\Sigma_{d}}$ for the left $\left(B_{L}\right)$ and right hemisphere $\left(B_{R}\right)$ separately, with *d* being the embedding dimension.

To obtain the final cortical parcellation, we applied *k* -means clustering to the feature vectors formed by the columns of $B_{L}$ and $B_{R}$ and varied the number of classes *k* from 21 to 200 to match the number of desired parcels in the parcellation. For each value of *k*, we ran *k*-means clustering with 50 different random initializations and selected the result that yielded the highest homogeneity of the average RSFC of all training subjects.

### 2.6 Evaluation metrics

#### 2.6.1 Resting-state functional connectivity (RSFC) homogeneity

Each subject’s RSFC was computed as the Pearson correlation of rsfMRI time series between all pairs of cortical vertices. A Fisher z-transform was subsequently performed to obtain z scores from the correlation values. Similar to the evaluation procedure in (Joshi et al., 2022; Schaefer et al., 2018), a parcel-wise homogeneity score *ρ*_*i*_ was computed as the averaged RSFC values within the *i*^th^ parcel. To obtain a global homogeneity measure for a single subject, a weighted average *ρ* of each parcel’s homogeneity scores was then computed by accounting for different cluster sizes:

(3) $$\rho =\sum_{i=1}^{N}\rho_{i}\frac{\left|V^{i}\right|}{\left|V\right|}$$

where $\left|V^{i}\right|$ is the number of vertices in parcel *i*, and *N* is the total number of parcels; $\left|V\right|$ is the total number of cortical vertices. For conciseness, we refer to this RSFC weighted average homogeneity as “homogeneity” hereafter. The homogeneity was computed for each test subject separately. To quantitatively compare the homogeneity of Untamed to that of other atlases, we performed a two-sided Wilcoxon signed rank test between each pair of parcellations, and an effect size was reported using Cohen’s *d* measure.

#### 2.6.2 Alignment with task contrasts

We also evaluated parcellation performance by comparing the parcellations to the group-average task contrast maps from the HCP 100 unrelated (U100) subjects (Barch et al., 2013) and the MDTB dataset (King et al., 2019; Zhi et al., 2022). The degree to which the regions delineated in a particular parcellation reflect functional specialization is assessed by computing the variance $\sigma_{i}^{2}$ of the task contrast within each parcel. The better the parcellation delineates regions of functional homogeneity, the lower the variance of task contrasts within each parcel. Similar to the procedure in (Schaefer et al., 2018; Joshi et al., 2022; Yan et al., 2023), the variance metric was first computed for each parcel, and then a weighted average was computed, accounting for parcel size differences, as:

(4) $$\sigma^{2}=\sum_{i=1}^{N}\sigma_{i}^{2}\frac{\left|V^{i}\right|}{\left|V\right|}$$

We refer to this weighted average task contrast variance as “task variance” hereafter. Note that we compute tasks variance separately for each contrast.

When comparing parcellation schemes, we calculated the difference between task variance between Untamed and its counterpart using a matched number of parcels. A negative value indicates Untamed has a lower variance, while a positive value indicates the counterpart has a lower variance. Statistical significance was evaluated using a two-sided Wilcoxon signed rank test between each pair of parcellations being compared.

#### 2.6.3 Agreement with selected cytoarchitectonic regions

**Fig. 2.**
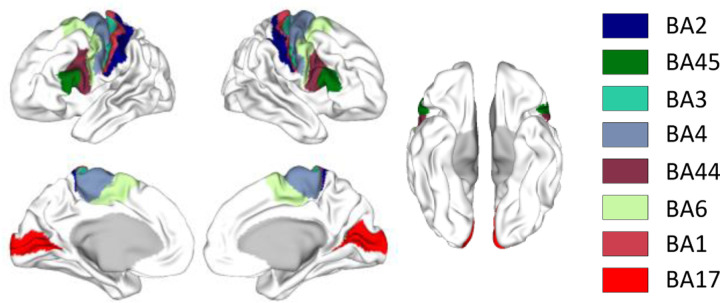
Selected architectonic areas in Brodmann atlas

We also evaluated the level of agreement between parcels obtained from each parcellation method and selected architectonic areas from the Brodmann atlas provided by HCP (https://balsa.wustl.edu/Wz8r). These regions of interest (ROIs) include primary somatosensory cortex (BA [1, 2, 3] (combining areas 3a and 3b)), primary motor cortex (BA 4), premotor cortex (BA 6), primary visual cortex (BA 17), and Broca’s area (BA [44 45]), as shown in Fig. 2. We used the parcellation evaluation script provided by (Arslan et al., 2018) to measure the agreement level between each architectonic area and the matched parcel(s). In brief, parcels that have a Dice coefficient higher than 0.5 with the same Brodmann ROI were merged together into a single parcel. Subsequently, the Dice coefficient was re-calculated between the consolidated parcel and the respective Brodmann ROI. We computed the difference between the Dice coefficients for Untamed parcellation and compared them to those of the other parcellation methods and atlases. Again, we used a two-sided Wilcoxon signed rank test for statistical testing.

### 2.7 Comparison with existing parcellations

We compared our Untamed parcellations to the extensive set of 17 atlases listed in Table 1. Because comparison of the evaluation metrics defined above is only meaningful when comparing parcellations of similar densities, in each case, we matched the number of parcels found using Untamed to the number in the left and right hemispheres for each baseline comparison.

For atlases that were originally constructed in a space different from HCP (e.g., MNI152, fsaverage), we used an atlas resampled onto the HCP fs_LR 32K surfaces provided either by the original authors or a third-party as listed in Table 1. We note that a certain percentage of cortical vertices on the resampled HCP surface for some baseline atlases may not be assigned to any parcel due to either methodological restriction or registration inaccuracy. In such cases, we discarded vertices in Untamed where labels were missing in the baseline atlases for a fair comparison. An extreme example of such cases is the atlas proposed by (Gordon et al., 2016), where approximately 30% of the vertices were unlabeled. For this case, we include both the original atlas (“Gordon”) with a substantial unlabeled cortical area and the refined atlas (“Gordon2”) provided by (Arslan et al., 2018), where the labeled regions are iteratively dilated until they cover the entire cortical surface. For the original two atlases proposed by (Yeo et al., 2011) that are spatially distributed across hemispheres with a relatively small number (7 and 17) of networks, we used the version provided in their GitHub repository (Table 1) where the distributed spatial networks were decomposed into local contiguous parcels (51 parcels for the 7 networks and 114 parcels for the 17 networks).

### 2.8 Ablation study of embedding methods

We also compared Untamed with the spectral clustering described in (Ng et al., 2001), which applies a *k* -means clustering to the most significant eigenvectors of the normalized graph Laplacian. All steps in the learning procedure are identical except for the graph embedding where we used NetMF. We applied both methods to the same graph constructed from NASCAR spatial maps as described in section 2.4. For both embedding methods, we set the spatial neighborhood constraint to be 25-hop, and the embedding dimension to be 100. Both of the embeddings were then separately supplied as input features for *k*-means clustering with 50 random initializations with a range of cluster numbers. We evaluated weighted average homogeneity on the averaged RSFC from the 500 training subjects in the HCP dataset. A two-sided Wilcoxon signed rank test was used to compare the homogeneity values obtained from the Laplacian eigenvectors used in spectral clustering and NetMF utilized in Untamed.

### 2.9 Ablation study of graph construction methods

In Untamed we constructed the graph using the correlation between features representing participation of each vertex in each of the NASCAR spatial network maps. Here, we conducted a comparison to the graph constructed from the widely used RSFC, which is the correlation between rsfMRI time series. Other steps are identical except for the construction of the graph. Parcellations generated from NASCAR spatial maps and RSFC were both subject to a spatial neighborhood constraint of 25-hop, and an embedding dimension of 100. We evaluated weighted average homogeneity on the averaged RSFC from the 500 training subjects in the HCP dataset. A two-sided Wilcoxon signed rank test was employed to statistically compare homogeneity values obtained from NASCAR spatial maps and RSFC.

### 2.10 Is there a (natural) optimal number of parcels that can be identified from rsfMRI data?

We used random parcellations as a null model to help us identify whether there is an optimal parcel number. We used the task variance, as described in section 2.6.2, as the performance metric. Specifically, we computed the ratio of the task variance obtained with Untamed to that from random parcellations. Random parcellations were generated through a region-growing process using the MNE-Python package (Gramfort, 2013). We computed this ratio and plotted it as a function of number of parcels (from 2 to 500 parcels per hemisphere). For each parcel number setting, 50 parcellation realizations were generated with different initialization seeds for both random and Untamed. The median of task variance across contrasts and trials was then computed for the HCP dataset and the MDTB dataset separately. The ratio plot was smoothed with a moving average with a window length of 5 for visualization.

## 3 Results

### 3.1 Resting-state functional connectivity (RSFC) homogeneity

**Fig. 3.**
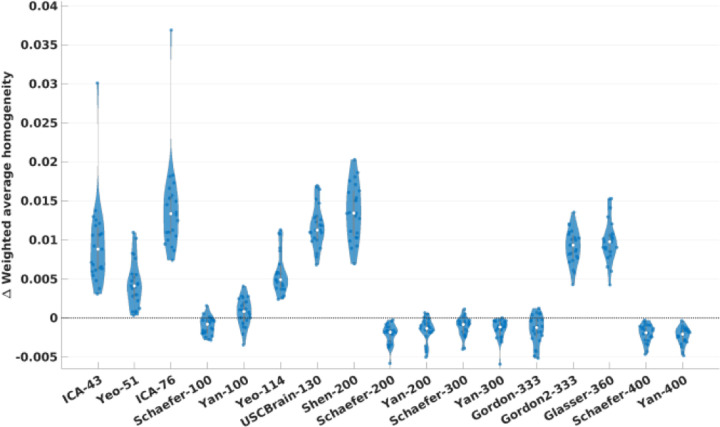
RSFC weighted average homogeneity evaluated on Yale test subjects. Each violin plot includes shows the subject-wise difference of RSFC weighted average homogeneity obtained with Untamed and the baseline counterpart (Untamed-baseline) for the 27 test subjects. A positive value indicates more homogeneity in the Untamed than baseline atlas, and vice versa. Statistics (effect size and *p*-values) are shown in Table 2.

Table 2 contains summary statistics comparing RSFC homogeneity between Untamed and each of the other atlases with matched numbers of parcels for the HCP and Yale test sets. The supplementary Fig. S1 (for HCP) and Fig. 3 (for Yale) show the subject-wise differences in homogeneity as violin plots. In these results, a positive Cohen’s *d* value indicates Untamed is more homogeneous than its counterpart and vice versa. When tested on the HCP data, Untamed achieves a clear improvement in homogeneity over all baseline atlases with statistical significance (*p* < 10^−3^) with the exception of Schaefer-400 (*p* = .62). Since these results may be biased as HCP data was also used to generate the Untamed atlases, we also include results for the Yale dataset. In this case, Untamed achieved higher homogeneities than 9 out of the 17 atlases, but was lower than the others, notably including all the Schafer atlases. However, in these cases, the effect sizes are relatively small (≤ .06). Because there are ~30% unlabeled vertices in Gordon, we excluded these vertices from Untamed to match the number of vertices when evaluating homogeneity. In this case, there is no significant difference. However, if we assign the missing vertices to the nearest parcel as described above (Gordon2) homogeneity is significantly higher for Untamed.

### 3.2 Alignment with task contrasts

**Fig. 4.**
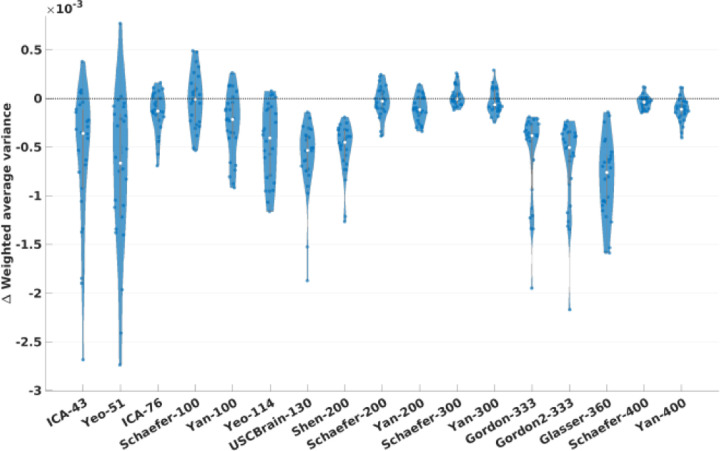
Weighted average task contrast variance evaluated on MDTB test subjects. Each violin plot represents the difference between the weighted average variance for each group-average contrast map with Untamed and the matched baseline (Untamed – baseline). A negative value indicates a lower average within-parcel variance in the Untamed atlas relative to the baseline atlas and vice versa. See text for discussion of statistical significance. See text for discussion of statistical significance.

The violin plot in Fig. 4 shows the distribution of the differences in task contrast variance (Eq. 4) between Untamed and the other atlases for the MDTB task dataset. An equivalent plot for the HCP task data is included in the supplementary Fig. S2. These plots show the distribution of paired differences between Untamed and each baseline atlas for each contrast. A negative value indicates an overall smaller contrast variance per parcel for Untamed relative to its counterpart. These results indicate a significantly (*p* < .001) lower variance for the HCP task data in the Untamed atlases relative to all other atlases, with the exception of the Schaefer-100 (*p* = .49). For the MDTB dataset, Untamed has a lower variance than the baseline atlases (*p* < .02) in 13 of 17 cases. In the remaining 4 cases (comparisons to Schaefer-100/200/300, Yan-300), there was no significant difference. Among the 26 task contrasts in the MDTB dataset, 15 had lower variance than Schaefer-100 and 200, 13 for Schaefer-300, and 16 for Yan-300. Notably, although Glasser-360 explicitly used HCP task fMRI data (in combination with structural data) in constructing the atlas, the variances computed on both the HCP and MDTB task data were significantly higher than those of Untamed.

**Fig. 5.**
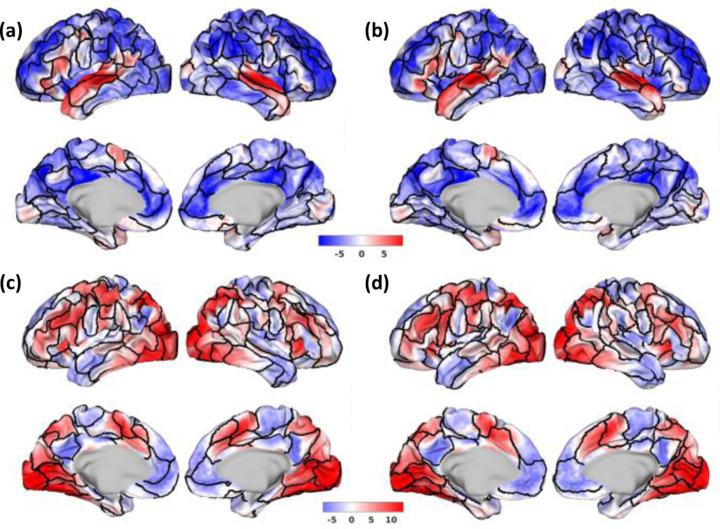
Example HCP group average task activation z-score maps for Untamed-100 and Schafer-100. First row: language_story contrast overlaid on boundaries (black) of (a) Untamed-100 (b) Schafer-100. Second row: relational_match contrast overlaid on (c) Untamed-100 (d) Schaefer-100. Weighted average variance of language_story contrast is 4.74 for Untamed-100, and 5.43 for Schaefer-100. Meanwhile, weighted average variance of relational_match contrast is 8.76 for Untamed-100, and 8.05 for Schaefer-100 (lower variance indicates better alignment)

It is also interesting to see that, for example, Untamed-100 and Schafer-100 have very similar overall quantitative performance, but their parcel boundaries are substantially different. As illustrated in Fig. 5 (a,b), where the parcel boundaries were overlaid on the HCP group average “story” contrast map in the language task, regions near Broca’s area and other auditory areas show a better alignment of Untamed parcels with the contrast compared to Schaefer’s. A counter-example is shown in Fig. 5 (c,d), where the relational_match contrast shows the better alignment of task-active regions with Schafer. We return to this observation in the discussion.

### 3.3 Agreement with selected cytoarchitectonic regions

**Fig. 6.**
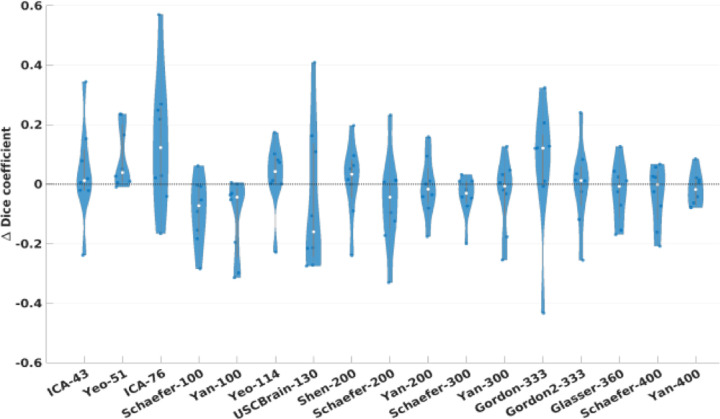
Alignment of parcels with 17 selected architectonic areas. Each violin plot represents the ROI-wise difference of the Dice coefficient between Untamed and baseline atlas (Untamed – baseline) across the 17 Brodmann areas after the matching and merging process described in Section 2.6.3.

Fig. 6 shows violin plots illustrating the difference in Dice coefficient between Untamed and baseline parcellations and the 17 corresponding Brodmann areas, as shown in Fig. 2. As described above, we use the script provided by (Arslan et al., 2018) to identify and merge parcels for each atlas that have significant overlap with the Brodmann areas prior to computing the Dice coefficient. In Fig. 6, positive values indicate larger Dice coefficients for Untamed relative to the baseline atlas. Among the 17 baseline parcellations, Untamed has a higher median Dice coefficient than 9 of the baselines. Since the number of regions is relatively small, in most cases, the differences between Untamed and the baseline atlases are not significant. The two exceptions are Yan-100, which outperforms Untamed-100 (*p* = .04), and Untamed-51, which outperforms Yeo-51 (*p* = .04). We note that among the atlases that show comparable performance to Untamed, both USCBrain-130 and Glasser-360 explicitly incorporate anatomical information, which may be expected to lead to better alignment with some Brodmann areas.

**Fig. 7.**
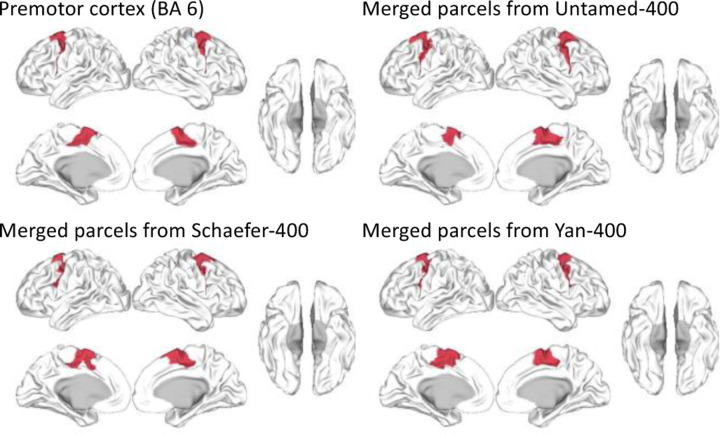
Red area denotes premotor area (BA 6) and merged parcels from Untamed, Schaefer and Yan, all with 400 parcels. Dice coefficient scores are 0.820, 0.796, and 0.799 respectively between the merged parcels with the ROI. Derived with script provided by (Arslan et al., 2018).

Fig. 7 shows a sample comparison between the Brodmann premotor cortex (BA 6) and the merged parcels of Untamed-400, Schaefer-400, and Yan-400. All atlases had similar Dice coefficients: Untamed (0.820), Schaefer (0.796), and Yan (0.799). Similar observations were found for most of the other Brodmann areas. Additional images comparing the merged parcels for these three atlases, along with their Dice scores, can be found in Fig. S3.

### 3.4 Ablation study of embedding methods

In Fig. S4, we compare parcellations obtained using graph node embedding (NetMF) prior to clustering as described above with results obtained using spectral clustering directly from the eigenvectors of the graph Laplacian (GLC). Again, we show the difference between Untamed and GLC-based atlases in RSFC weighted average homogeneity per HCP test. Among the 11 different numbers of parcels tested, NetMF-based Untamed outperforms the GLC-based one in 7 cases with large effect sizes. In 2 cases (100, 130), there is no statistically significant difference and in the remaining 2 cases (114, 200) GLC is statistically significantly better (*p* < .05), but the effect size is small. These results support the use of NetMF embedding in place of the more standard GLC approach.

### 3.5 Ablation study of graph construction methods

We also explored the effect of graph construction as described in Section 2.9 by comparing results using the NASCAR-based adjacency matrix with that computed directly using Pearson correlation between rsfMRI time series. All other aspects of processing were identical. Fig. S5 shows the difference in RSFC weighted average homogeneity between NASCAR-based and Pearson-based adjacency matrices. It is evident that the parcellations generated from the NASCAR-based adjacency matrix substantially outperform those generated from Pearson-based adjacency in all cases (*p* < .00001). This demonstrates an advantage of using the results of NASCAR tensor decomposition to identify spatial networks over that using the Pearson correlation between rsfMRI time series.

### 3.6 Is there an optimal number of parcels?

**Fig. 8.**
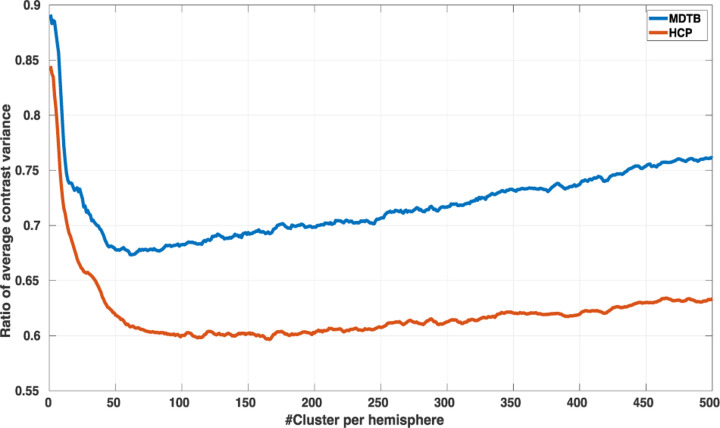
The ratio of median contrast variance across all task contrasts and all trials between Untamed and random parcellations constructed using region growing for the MDTB and HCP task datasets.

To explore this question, we compared how well the Untamed atlas identified regions of relatively homogenous functional activity, as reflected in the task maps, compared to random parcellations with an equal number of regions. Fig. 8 shows the ratio of variance for the HCP and MDTB task datasets between Untamed and random parcellations. The curve obtained with HCP data is different from that with MDTB, possibly because of the different battery of fMRI tasks and contrasts employed. There is a distinct shallow minimum in the ratio plot in the MDTB data at ~61 parcels per hemisphere. The curve associated with the HCP dataset has a less clear trend where the optimal number of parcels lies between 100 and 150.

## 4 Discussion

**Fig. 9.**
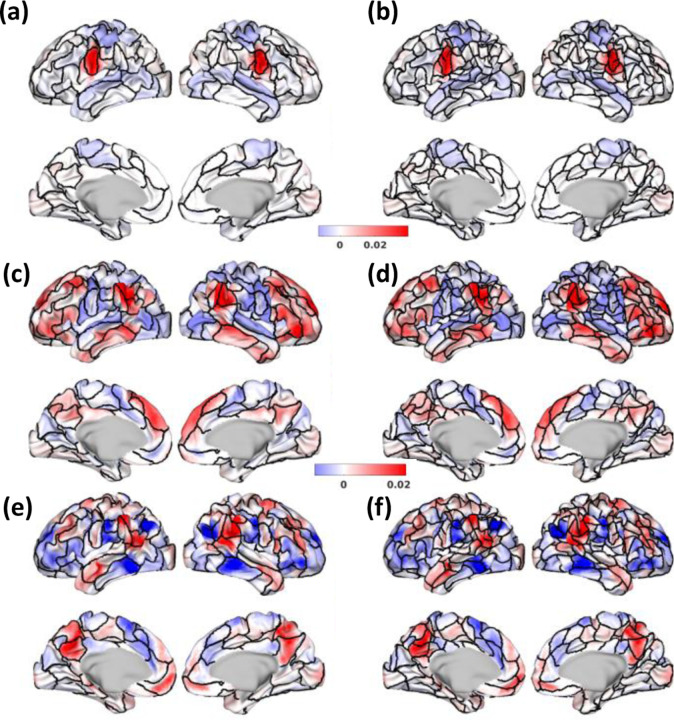
(a) and (b) depict NASCAR somatomotor sub-network with (a) Untamed-100 (b) Untamed-300 parcel boundaries; (c) and (d) display NASCAR default mode sub-network with (c) Untamed-100 and (d) Untamed-300; (e) and (f) display NASCAR ventral attentional network with (e) Untamed-100 and Untamed-300.

In this paper we introduce “Untamed”, a novel cortical parcellation scheme developed from population resting-state fMRI data. This scheme constructs spatially disjoint parcels by leveraging the spatially overlapped brain networks identified by NASCAR tensor decomposition (Li et al., 2023, 2021). Fig. 9 shows a visual representation of both the 100 and 300 parcel versions of Untamed superimposed on the somatomotor sub-network, the default mode sub-network, and the ventral attention network of NASCAR. A key observation is that the parcel boundaries in Untamed align fairly closely with the transition between activated and deactivated regions in the NASCAR networks. Consequently, Untamed effectively bridges the gap between the spatially overlapping brain networks identified by NASCAR and the pursuit of spatially distinct brain parcellation.

**Fig. 10.**
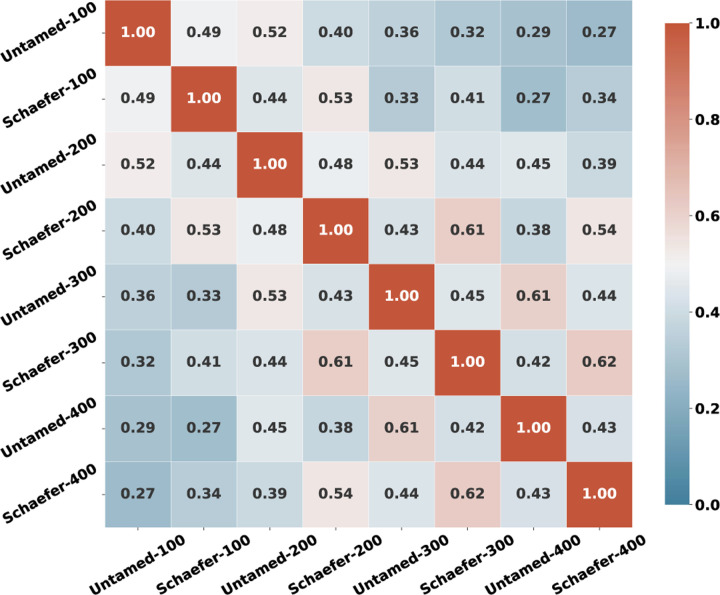
Adjusted rand index (ARI) between Untamed and Schaefer series across different cluster numbers.

We compared Untamed to an extensive list of popular atlases and parcellations methods as listed in Table 1. The results shown for homogeneity of functional connectivity (Fig. 3), task-contrast variance (Fig. 4) and alignment with Brodmann areas (Fig. 6) indicate that among existing atlases, the Yan and Schaefer atlases perform particularly well. Untamed exhibits overall similar performance (with matched number of parcels) to these two parcellation methods. Interestingly, while overall performance is quite similar, the actual parcellations are quite different. This is illustrated in Fig. 9 which shows examples where Untamed and then Schaefer respectively show a better match to task activation maps for two different task contrasts. To explore this difference quantitatively we include in Fig. 10 the averaged adjusted rand index (ARI) indicating the overlap between matched parcels among pairs of atlases. ARI is a metric for assessing similarity between two cluster sets, accomplished by considering all pairs of samples and counting the number of pairs assigned to the same or different labels. ). Parcels are matched using the Hungarian algorithm. Values range from 0 (random labeling) to 1 (identical clustering up to a permutation). A visual comparison between the two atlas series can be found in Fig. S7. The highest ARI between any two different parcellation schemes is 0.61 (between Schaefer-300 and Untamed-300 and also between Schaefer-200 and Untamed-300). Even within parcellation approaches the highest ARI is only 0.62 (Schaefer-300 and Schaefer-400).

**Fig. 11.**
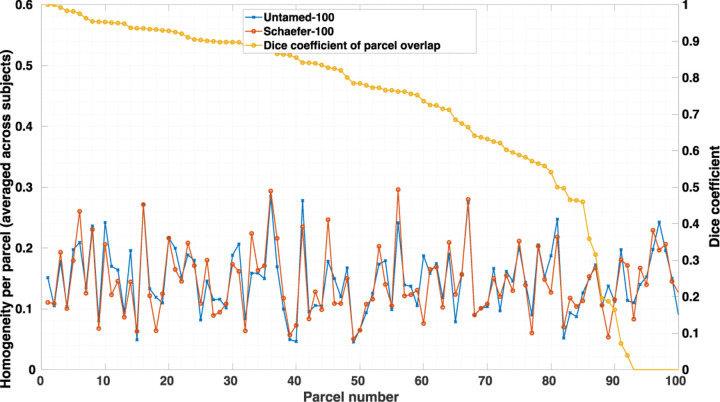
Parcel-wise RSFC homogeneity scores (averaged across subjects) in Untamed-100 and Schaefer-100 computed on Yale rsfMRI test subjects. Parcels are matched across atlases using the Hungarian matching algorithm. Also shown are the Dice ceofficients (rank ordered) between matched parcels.

The difference in parcellations between atlases with similar performance seems to indicate that there may exist parcellations that exceed the performance of both by combining the best facets of each. Further support for this conjecture is shown in Fig 11 where we show the RSFC homogeneity of Untamed-100 and Schaefer-100 as well as their Dice coefficient for each of the Hugarian-algorithm matched parcels. The per-parcel homogeneity scores between the two are much more consistent than Untamed-100 and Random-100 (random parcellation from region growing as described in section 2.10) as depicted in Fig. S6. However, it appears that there is still room for further improvement with respect to the Untamed and Schaefer results.

It is important to note that both the Schaefer and Untamed schemes are based on rsfMRI; however, they were generated using distinct acquisition protocols and independent subjects. The publicly released Schaefer atlases, which were used in our experiments, were derived from approximately 1500 subjects in the Genome Project dataset (GSP), whereas our atlases were generated from an independent set of 500 subjects from the Human Connectome Project (HCP). Both datasets consist of young adults (GSP: 18–35 yrs, HCP: 22–35 yrs). In addition to the different subjects and data acquisitions, Schaefer utilized an entirely different methodology (based on a Markov Random Field model) and uses a distinct feature (RSFC). Exploring the application of the Untamed methodology using the GSP data may offer further insights into the similarity and differences between these two methodologies.

**Fig. 12.**
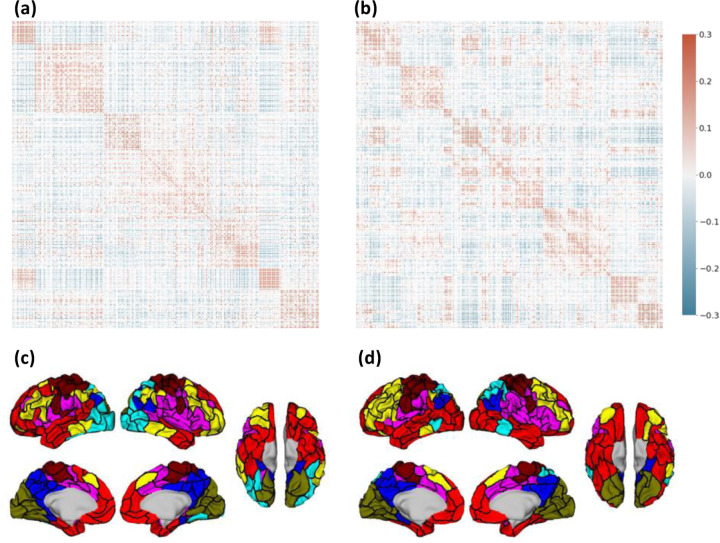
RSFC based on (a) Untamed-200 and (b) Schaefer-200 (averaged across subjects) on Yale rsfMRI test subjects after global signal regression. Both clustered to 7 networks as in (Yeo et al., 2011) using spectral clustering. (c) and (d) shows all parcels of Untamed-200 and Shaefer-200 assigned network colors

As a final comparison we computed the RSFC between parcels for the Untamed-200 and Schaefer-200 atlases. We used the Yale rsfMRI dataset and performed a global signal regression before computing the Pearson correlation between parcels on signals formed by averaging the rsfMRI signal across each parcel, Fig. 12. We then used an automated spectral clustering (Ng et al., 2001) method to identify 7 networks consisting of groups of parcels that exhibit the most similarity in their connectivity patterns. The ordered RSFC matrices and the corresponding set of 7 networks (color coded) for each atlas are shown in Fig 12.

The existence of a large number of brain atlases raises the question of which to select for a particular purpose. In general, a cortical parcellation should aim to segregate the cortex into distinct regions, with the objective of maximizing the similarity of connectivity patterns, task activation, and/or anatomical measures within each (Zhi et al., 2022). The literature commonly employs atlases for data dimensionality reduction, although the selection process often appears somewhat arbitrary and may rely on certain assumptions, such as their suitability across various populations (Bryce et al., 2021). We suggest that the choice of atlas should be guided by the specific objectives of the study. For example, for analysis of resting data across different populations, selection of an atlas with the highest RSFC homogeneity would likely be the optimal choice. For analysis of event-related data, atlases that perform best on task-based evaluation may be preferable.

A second important question is the number of parcels that should be used in the atlas (Schaefer et al., 2018; Yan et al., 2023). Our study of the variance of task contrasts with Untamed vs. random parcellations in Fig. 8 shows that the maximum advantage over random parcellations occurs between 50 and 150 parcels per hemisphere, depending on the dataset used. Beyond 150, the advantage of using a principled approach over random seed-based region-growing slowly diminishes. This suggests that increasing parcellation density will at some point offer diminishing returns in terms of reliably defining meaningful subdivisions.

In the ablation studies we performed, we saw a clear advantage to using network embedding with NetMF rather than directly clustering using a spectral decomposition (Fig. S4). We note that there are other parcellation approaches that use spectral clustering, for example (Craddock et al., 2012), where the final clusters were generated through discretization (Yu and Shi, 2003). While this approach shares similiarites to Untamed, it limites the range of the number of parcels.

The other ablation study looked at the effect of replacing feature-vectors computed from the tensor-decomposition of the rsfMRI data in Untamed with direct calculation of Pearson correlation of time-series between cortical surface vertices, Fig. S5. These results showed improved performance in terms of RSFC homogeneity for all parcel numbers.

Untamed consists of three discrete steps once the rsfMRI are processed and spatially aligned: (i) BrainSync synchronization of rsfMRI data, (ii) NASCAR-based tensor-decomposition, and (iii) NetMF-based graph embedding and *k* -means clustering. Of these, NASCAR could be time consuming depending on the number of subjects and the density of the cortical surface mesh used to define the graph nodes. The third step is fast and results can be computed with a few minutes on a personal computer.

Despite our efforts to ensure comprehensive and unbiased evaluation comparisons, there are inherent disparities in our study. Untamed schemes were derived using the HCP dataset in fs_LR 32K surface space, while we utilized the projected atlases in other spaces to match the fs_LR 32K space. The registration process can potentially impact performance. We have attempted to avoid biases in evaluation by basing most of the evaluation presented in the body of the paper on dataset sets collected independently, and with different acquisition protocols, from the HCP data used to generate the atlases.

To further enhance the parcellations obtained using the framework described here, alternative methods for factorization of the NetMF matrix (Eq. 2) could be explored. Our current methodology follows the practice in (Qiu et al., 2018), which utilizes the left singular vectors and singular values of the NetMF matrix to construct low-dimensional embeddings. However, other factorization techniques, such as stochastic matrix factorization, which can incorporate weighting for different vertices, may potentially yield improved results (Levy and Goldberg, 2014). Another potential avenue for improvement in our study is to extend beyond group parcellations and consider individual variability. The optimal division of the cerebral cortex can significantly differ among subjects and recent studies has explored individualized parcellations (Chong et al., 2017; Kong et al., 2019). Individualized parcellations could be realized using the Untamed framework by leveraging information on the subject’s participation level within each of the networks, identified in the NASCAR tensor-decomposition, when defining the graph from which the embedding is subsequently computed.

## Supplementary Material

Supplement 1

## Figures and Tables

**Table 1.** List of atlases included in the comparison. Type denotes the modality of data the atlas is constructed from. “Functional”: the atlas is only based on fMRI; “Hybrid”: the atlas is constructed from both functional MRI and anatomical information.

Name	Type	# Clusters	Reference	Atlas Source
ICA-43 ICA-76	Functional	43 (L: 21, R: 22) 76 (L: 36, R: 40)	https://humanconnectome.org/storage/app/media/documentation/s1200/HCP1200-DenseConnectome+PTN+Appendix-July2017.pdf	https://db.humanconnectome.org/data/projects/HCP_1200
Yeo-51 Yeo-114	Functional	51 (L: 26, R: 25) 114 (L: 57, R: 57)	(Yeo et al., 2011)	https://github.com/ThomasYeoLab/CBIG/tree/master/stable_projects/brain_parcellation
USCBrain	Hybrid	130 (L: 65, R: 65)	(Joshi et al., 2022)	https://github.com/ajoshiusc/bfp/blob/main/supp_data/USCBrain_grayordinate_labels_clean.mat
Shen	Functional	200 (L: 102, R: 98)	(Shen et al., 2013)	https://biomedia.doc.ic.ac.uk/brain-parcellation-survey/
Schaefer-100/200/300/400	Functional	100 (L: 50, R: 50) 200 (L: 100, R: 100) 300 (L: 150, R: 150) 400 (L: 200, R: 200)	(Schaefer et al., 2018)	https://github.com/ThomasYeoLab/CBIG/tree/master/stable_projects/brain_parcellation
Yan-100/200/300/400	Functional	100 (L: 50, R: 50) 200 (L: 100, R: 100) 300 (L: 150, R: 150) 400 (L: 200, R: 200)	(Yan et al., 2023)	https://github.com/ThomasYeoLab/CBIG/tree/master/stable_projects/brain_parcellation
Gordon Gordon2	Functional	333 (L: 161, R: 172)	(Gordon et al., 2016) (Arslan et al., 2018)	https://balsa.wustl.edu/WK71 https://biomedia.doc.ic.ac.uk/brain-parcellation-survey/
Glasser	Hybrid	360 (L: 180, R: 180)	(Glasser et al., 2016)	https://balsa.wustl.edu/study/show/RVVG

**Table 2.** Effect sizes (measured by Cohen’s *d*) and *p*-values of subject-wise RSFC homogeneity differences between Untamed and baseline atlases on HCP and Yale datasets. A positive Cohen’s *d* indicates that Untamed introduces a higher RSFC homogeneity than the baseline counterpart, and vice versa. The *p* -values are uncorrected.

Name-number of parcels	HCP rsfMRI Cohen’s *d* (𝒑-value)	Yale rsfMRI Cohen’s *d* (𝒑-value)
ICA-43	0.105 (*p* < 10^−3^)	0.306 (*p* < 10^−3^)
Yeo-51	0.143 (*p* < 10^−3^)	0.137(*p* < 10^−3^)
ICA-76	0.198 (*p* < 10^−3^)	0.445 (*p* < 10^−3^)
Schaefer-100	0.014 (*p* < 10^−3^)	−0.031 (*p* < 10^−3^)
Yan-100	0.080 (*p* < 10^−3^)	0.020 (*p* = 0.07)
Yeo-114	0.152 (*p* < 10^−3^)	0.169 (*p* < 10^−3^)
USCBrain-130	0.344 (*p* < 10^−3^)	0.352 (*p* < 10^−3^)
Shen-200	0.423 (*p* < 10^−3^)	0.398 (*p* < 10^−3^)
Schaefer-200	0.021 (*p* < 10^−3^)	−0.060 (*p* < 10^−3^)
Yan-200	0.030 (*p* < 10^−3^)	−0.043 (*p* < 10^−3^)
Schaefer-300	0.025 (*p* < 10^−3^)	−0.031 (*p* < 10^−3^)
Yan-300	0.027 (*p* < 10^−3^)	−0.039 (*p* < 10^−3^)
Gordon-333	0.027 (*p* < 10^−3^)	−0.039 (*p* = .002)
Gordon2–333	0.299 (*p* < 10^−3^)	0.255 (*p* < 10^−3^)
Glasser-360	0.301 (*p* < 10^−3^)	0.277 (*p* < 10^−3^)
Schaefer-400	0.000 (*p* = 0.62)	−0.053 (*p* < 10^−3^)
Yan-400	0.007 (*p* < 10^−3^)	−0.060 (*p* < 10^−3^)

## References

[R1] Akrami H., Joshi A.A., Li J., Leahy R.M., 2019. Group-wise alignment of resting fMRI in space and time, in: Angelini E.D., Landman B.A. (Eds.), SPIE Medical Imaging 2019: Image Processing. Presented at the Image Processing, San Diego, United States, p. 103. 10.1117/12.2512564

[R2] Allen E.A., Damaraju E., Plis S.M., Erhardt E.B., Eichele T., Calhoun V.D., 2014. Tracking Whole-Brain Connectivity Dynamics in the Resting State. Cerebral Cortex 24, 663–676. 10.1093/cercor/bhs352 23146964 PMC3920766

[R3] Andrews-Hanna J.R., Reidler J.S., Sepulcre J., Poulin R., Buckner R.L., 2010. Functional-Anatomic Fractionation of the Brain’s Default Network. Neuron 65, 550–562. 10.1016/j.neuron.2010.02.005 20188659 PMC2848443

[R4] Andrews‐Hanna J.R., Smallwood J., Spreng R.N., 2014. The default network and self‐generated thought: component processes, dynamic control, and clinical relevance. Annals of the New York Academy of Sciences 1316, 29–52. 10.1111/nyas.12360 24502540 PMC4039623

[R5] Arslan S., Ktena S.I., Makropoulos A., Robinson E.C., Rueckert D., Parisot S., 2018. Human brain mapping: A systematic comparison of parcellation methods for the human cerebral cortex. NeuroImage 170, 5–30. 10.1016/j.neuroimage.2017.04.014 28412442

[R6] Barch D.M., Burgess G.C., Harms M.P., Petersen S.E., Schlaggar B.L., Corbetta M., Glasser M.F., Curtiss S., Dixit S., Feldt C., Nolan D., Bryant E., Hartley T., Footer O., Bjork J.M., Poldrack R., Smith S., Johansen-Berg H., Snyder A.Z., Van Essen D.C., 2013. Function in the human connectome: Task-fMRI and individual differences in behavior. NeuroImage 80, 169–189. 10.1016/j.neuroimage.2013.05.033 23684877 PMC4011498

[R7] Beckmann C.F., DeLuca M., Devlin J.T., Smith S.M., 2005. Investigations into resting-state connectivity using independent component analysis. Phil. Trans. R. Soc. B 360, 1001–1013. 10.1098/rstb.2005.1634 16087444 PMC1854918

[R8] Biswal B., Zerrin Yetkin F., Haughton V.M., Hyde J.S., 1995. Functional connectivity in the motor cortex of resting human brain using echo-planar mri. Magn. Reson. Med. 34, 537–541. 10.1002/mrm.1910340409 8524021

[R9] Brodmann K., 1909. Vergleichende Lokalisationslehre der Großhirnrinde : in ihren Prinzipien dargestellt auf Grund des Zellenbaues.

[R10] Bryce N.V., Flournoy J.C., Guassi Moreira J.F., Rosen M.L., Sambook K.A., Mair P., McLaughlin K.A., 2021. Brain parcellation selection: An overlooked decision point with meaningful effects on individual differences in resting-state functional connectivity. NeuroImage 243, 118487. 10.1016/j.neuroimage.2021.118487 34419594 PMC8629133

[R11] Buckner R.L., DiNicola L.M., 2019. The brain’s default network: updated anatomy, physiology and evolving insights. Nat Rev Neurosci 20, 593–608. 10.1038/s41583-019-0212-7 31492945

[R12] Calhoun V.D., Adali T., Pearlson G.D., Pekar J.J., 2001. A method for making group inferences from functional MRI data using independent component analysis. Hum. Brain Mapp. 14, 140–151. 10.1002/hbm.1048 11559959 PMC6871952

[R13] Chong M., Bhushan C., Joshi A.A., Choi S., Haldar J.P., Shattuck D.W., Spreng R.N., Leahy R.M., 2017. Individual parcellation of resting fMRI with a group functional connectivity prior. NeuroImage 156, 87–100. 10.1016/j.neuroimage.2017.04.054 28478226 PMC5774339

[R14] Cichocki A., Mandic D., De Lathauwer L., Zhou G., Zhao Q., Caiafa C., Phan H.A., 2015. Tensor Decompositions for Signal Processing Applications: From two-way to multiway component analysis. IEEE Signal Process. Mag. 32, 145–163. 10.1109/MSP.2013.2297439

[R15] Craddock R.C., James G.A., Holtzheimer P.E., Hu X.P., Mayberg H.S., 2012. A whole brain fMRI atlas generated via spatially constrained spectral clustering. Hum. Brain Mapp. 33, 1914–1928. 10.1002/hbm.21333 21769991 PMC3838923

[R16] Eickhoff S.B., Yeo B.T.T., Genon S., 2018. Imaging-based parcellations of the human brain. Nat Rev Neurosci 19, 672–686. 10.1038/s41583-018-0071-7 30305712

[R17] Esteban O., Markiewicz C.J., Blair R.W., Moodie C.A., Isik A.I., Erramuzpe A., Kent J.D., Goncalves M., DuPre E., Snyder M., Oya H., Ghosh S.S., Wright J., Durnez J., Poldrack R.A., Gorgolewski K.J., 2019. fMRIPrep: a robust preprocessing pipeline for functional MRI. Nat Methods 16, 111–116. 10.1038/s41592-018-0235-4 30532080 PMC6319393

[R18] Fan L., Zhong Q., Qin J., Li N., Su J., Zeng L., Hu D., Shen H., 2021. Brain parcellation driven by dynamic functional connectivity better capture intrinsic network dynamics. Hum Brain Mapp 42, 1416–1433. 10.1002/hbm.25303 33283954 PMC7927310

[R19] Glasser M.F., Coalson T.S., Robinson E.C., Hacker C.D., Harwell J., Yacoub E., Ugurbil K., Andersson J., Beckmann C.F., Jenkinson M., Smith S.M., Van Essen D.C., 2016. A multi-modal parcellation of human cerebral cortex. Nature 536, 171–178. 10.1038/nature18933 27437579 PMC4990127

[R20] Glasser M.F., Sotiropoulos S.N., Wilson J.A., Coalson T.S., Fischl B., Andersson J.L., Xu J., Jbabdi S., Webster M., Polimeni J.R., Van Essen D.C., Jenkinson M., 2013. The minimal preprocessing pipelines for the Human Connectome Project. NeuroImage 80, 105–124. 10.1016/j.neuroimage.2013.04.127 23668970 PMC3720813

[R21] Gopinath K., Desrosiers C., Lombaert H., 2019. Graph Convolutions on Spectral Embeddings for Cortical Surface Parcellation. Medical Image Analysis 54, 297–305. 10.1016/j.media.2019.03.012 30974398

[R22] Gordon E.M., Laumann T.O., Adeyemo B., Huckins J.F., Kelley W.M., Petersen S.E., 2016. Generation and Evaluation of a Cortical Area Parcellation from Resting-State Correlations. Cereb. Cortex 26, 288–303. 10.1093/cercor/bhu239 25316338 PMC4677978

[R23] Gramfort A., 2013. MEG and EEG data analysis with MNE-Python. Front. Neurosci. 7. 10.3389/fnins.2013.00267 PMC3872725 24431986

[R24] Han M., Yang G., Li H., Zhou S., Xu B., Jiang J., Men W., Ge J., Gong G., Liu H., Gao J.-H., 2020. Individualized Cortical Parcellation Based on Diffusion MRI Tractography. Cerebral Cortex 30, 3198–3208. 10.1093/cercor/bhz303 31814022

[R25] Harrison S.J., Bijsterbosch J.D., Segerdahl A.R., Fitzgibbon S.P., Farahibozorg S.-R., Duff E.P., Smith S.M., Woolrich M.W., 2020. Modelling subject variability in the spatial and temporal characteristics of functional modes. NeuroImage 222, 117226. 10.1016/j.neuroimage.2020.117226 32771617 PMC7779373

[R26] He R., Gopinath K., Desrosiers C., Lombaert H., 2020. Spectral Graph Transformer Networks for Brain Surface Parcellation, in: 2020 IEEE 17th International Symposium on Biomedical Imaging (ISBI). Presented at the 2020 IEEE 17th International Symposium on Biomedical Imaging (ISBI), IEEE, Iowa City, IA, USA, pp. 372–376. 10.1109/ISBI45749.2020.9098737

[R27] Joshi A.A., Choi S., Liu Y., Chong M., Sonkar G., Gonzalez-Martinez J., Nair D., Wisnowski J.L., Haldar J.P., Shattuck D.W., Damasio H., Leahy R.M., 2022. A hybrid high-resolution anatomical MRI atlas with sub-parcellation of cortical gyri using resting fMRI. Journal of Neuroscience Methods 374, 109566. 10.1016/j.jneumeth.2022.109566 35306036 PMC9302382

[R28] Joshi A.A., Chong M., Li J., Choi S., Leahy R.M., 2018. Are you thinking what I’m thinking? Synchronization of resting fMRI time-series across subjects. NeuroImage 172, 740–752. 10.1016/j.neuroimage.2018.01.058 29428580 PMC6338442

[R29] King M., Hernandez-Castillo C.R., Poldrack R.A., Ivry R.B., Diedrichsen J., 2019. Functional boundaries in the human cerebellum revealed by a multi-domain task battery. Nat Neurosci 22, 1371–1378. 10.1038/s41593-019-0436-x 31285616 PMC8312478

[R30] Kolda T.G., Bader B.W., 2009. Tensor Decompositions and Applications. SIAM Rev. 51, 455–500. 10.1137/07070111X

[R31] Kong R., Li J., Orban C., Sabuncu M.R., Liu H., Schaefer A., Sun N., Zuo X.-N., Holmes A.J., Eickhoff S.B., Yeo B.T.T., 2019. Spatial Topography of Individual-Specific Cortical Networks Predicts Human Cognition, Personality, and Emotion. Cerebral Cortex 29, 2533–2551. 10.1093/cercor/bhy123 29878084 PMC6519695

[R32] Kuhn H.W., 1955. The Hungarian method for the assignment problem. Naval Research Logistics 2, 83–97. 10.1002/nav.3800020109

[R33] Lee K., Horien C., O’Connor D., Garand-Sheridan B., Tokoglu F., Scheinost D., Lake E.M.R., Constable R.T., 2022. Arousal impacts distributed hubs modulating the integration of brain functional connectivity. NeuroImage 258, 119364. 10.1016/j.neuroimage.2022.119364 35690257 PMC9341222

[R34] Levy O., Goldberg Y., 2014. Neural Word Embedding as Implicit Matrix Factorization. Presented at the Advances in Neural Information Processing Systems.

[R35] Li J., Liu Y., Wisnowski J.L., Leahy R.M., 2023. Identification of overlapping and interacting networks reveals intrinsic spatiotemporal organization of the human brain. NeuroImage 270, 119944. 10.1016/j.neuroimage.2023.119944 36801371 PMC10092006

[R36] Li J., Wisnowski J.L., Joshi A.A., Leahy R.M., 2021. Robust brain network identification from multi-subject asynchronous fMRI data. NeuroImage 227, 117615. 10.1016/j.neuroimage.2020.117615 33301936 PMC7983296

[R37] Ng A., Jordan M., Weiss Y., 2001. On Spectral Clustering: Analysis and an algorithm, in: Advances in Neural Information Processing Systems.

[R38] Perozzi B., Al-Rfou R., Skiena S., 2014. DeepWalk: online learning of social representations, in: Proceedings of the 20th ACM SIGKDD International Conference on Knowledge Discovery and Data Mining. Presented at the KDD ‘14: The 20th ACM SIGKDD International Conference on Knowledge Discovery and Data Mining, ACM, New York New York USA, pp. 701–710. 10.1145/2623330.2623732

[R39] Qiu J., Dong Y., Ma H., Li J., Wang K., Tang J., 2018. Network Embedding as Matrix Factorization: Unifying DeepWalk, LINE, PTE, and node2vec, in: Proceedings of the Eleventh ACM International Conference on Web Search and Data Mining. Presented at the WSDM 2018: The Eleventh ACM International Conference on Web Search and Data Mining, ACM, Marina Del Rey CA USA, pp. 459–467. 10.1145/3159652.3159706

[R40] Schaefer A., Kong R., Gordon E.M., Laumann T.O., Zuo X.-N., Holmes A.J., Eickhoff S.B., Yeo B.T.T., 2018. Local-Global Parcellation of the Human Cerebral Cortex from Intrinsic Functional Connectivity MRI. Cerebral Cortex 28, 3095–3114. 10.1093/cercor/bhx179 28981612 PMC6095216

[R41] Shen X., Tokoglu F., Papademetris X., Constable R.T., 2013. Groupwise whole-brain parcellation from resting-state fMRI data for network node identification. NeuroImage 82, 403–415. 10.1016/j.neuroimage.2013.05.081 23747961 PMC3759540

[R42] Smith S.M., Hyvärinen A., Varoquaux G., Miller K.L., Beckmann C.F., 2014. Group-PCA for very large fMRI datasets. NeuroImage 101, 738–749. 10.1016/j.neuroimage.2014.07.051 25094018 PMC4289914

[R43] Tzourio-Mazoyer N., Landeau B., Papathanassiou D., Crivello F., Etard O., Delcroix N., Mazoyer B., Joliot M., 2002. Automated Anatomical Labeling of Activations in SPM Using a Macroscopic Anatomical Parcellation of the MNI MRI Single-Subject Brain. NeuroImage 15, 273–289. 10.1006/nimg.2001.0978 11771995

[R44] Van Essen D.C., Ugurbil K., Auerbach E., Barch D., Behrens T.E.J., Bucholz R., Chang A., Chen L., Corbetta M., Curtiss S.W., Della Penna S., Feinberg D., Glasser M.F., Harel N., Heath A.C., Larson-Prior L., Marcus D., Michalareas G., Moeller S., Oostenveld R., Petersen S.E., Prior F., Schlaggar B.L., Smith S.M., Snyder A.Z., Xu J., Yacoub E., 2012. The Human Connectome Project: A data acquisition perspective. NeuroImage 62, 2222–2231. 10.1016/j.neuroimage.2012.02.018 22366334 PMC3606888

[R45] Varoquaux G., Sadaghiani S., Pinel P., Kleinschmidt A., Poline J.B., Thirion B., 2010. A group model for stable multi-subject ICA on fMRI datasets. NeuroImage 51, 288–299. 10.1016/j.neuroimage.2010.02.010 20153834

[R46] Yan X., Kong R., Xue A., Yang Q., Orban C., An L., Holmes A.J., Qian X., Chen J., Zuo X.-N., Zhou J.H., Fortier M.V., Tan A.P., Gluckman P., Chong Y.S., Meaney M.J., Bzdok D., Eickhoff S.B., Yeo B.T.T., 2023. Homotopic local-global parcellation of the human cerebral cortex from resting-state functional connectivity. NeuroImage 120010. 10.1016/j.neuroimage.2023.120010 36918136 PMC10212507

[R47] Yeo T., Krienen F.M., Sepulcre J., Sabuncu M.R., Lashkari D., Hollinshead M., Roffman J.L., Smoller J.W., Zöllei L., Polimeni J.R., Fischl B., Liu H., Buckner R.L., 2011. The organization of the human cerebral cortex estimated by intrinsic functional connectivity. Journal of Neurophysiology 106, 1125–1165. 10.1152/jn.00338.2011 21653723 PMC3174820

[R48] Yu Shi, 2003. Multiclass spectral clustering, in: Proceedings Ninth IEEE International Conference on Computer Vision. Presented at the ICCV 2003: 9th International Conference on Computer Vision, IEEE, Nice, France, pp. 313–319 vol.1. 10.1109/ICCV.2003.1238361

[R49] Zhang W., Wang Y., 2019. Geometric Brain Surface Network for Brain Cortical Parcellation, in: Zhang D., Zhou L., Jie B., Liu M. (Eds.), Graph Learning in Medical Imaging, Lecture Notes in Computer Science. Springer International Publishing, Cham, pp. 120–129. 10.1007/978-3-030-35817-4_15 PMC8048406 33870335

[R50] Zhi D., King M., Hernandez‐Castillo C.R., Diedrichsen J., 2022. Evaluating brain parcellations using the distance‐controlled boundary coefficient. Human Brain Mapping 43, 3706–3720. 10.1002/hbm.25878 35451538 PMC9294308

