# Supplementary material for: Graph Learning for Cortical Parcellation from Tensor Decompositions of Resting-State fMRI
Source: bioRxiv. 2024 Jan 17:2024.01.05.574423. Preprint. [Version 2] doi: 10.1101/2024.01.05.574423 (PMC10802375; doi:10.1101/2024.01.05.574423)
Supplement: Supplementary file 1 [file media-1.pdf]

Supplementary materials

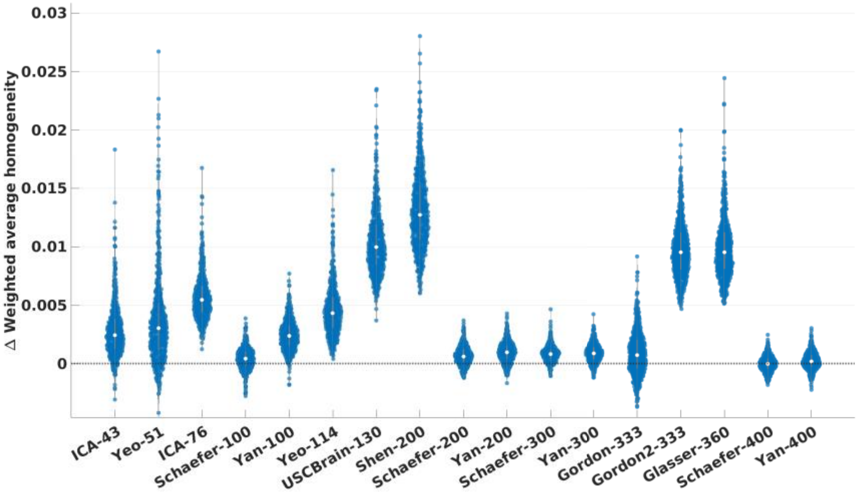

Fig. S1. RSFC weighted average homogeneity evaluated on HCP test subjects. Each violin plot shows the subject-wise difference of RSFC weighted average homogeneity between Untamed and the baseline counterpart (Untamed-baseline) for the 500 test subjects. A positive value indicates more homogeneity in the Untamed than baseline atlas, and vice versa. Statistics (effect size and p-values) are shown in Table 2.

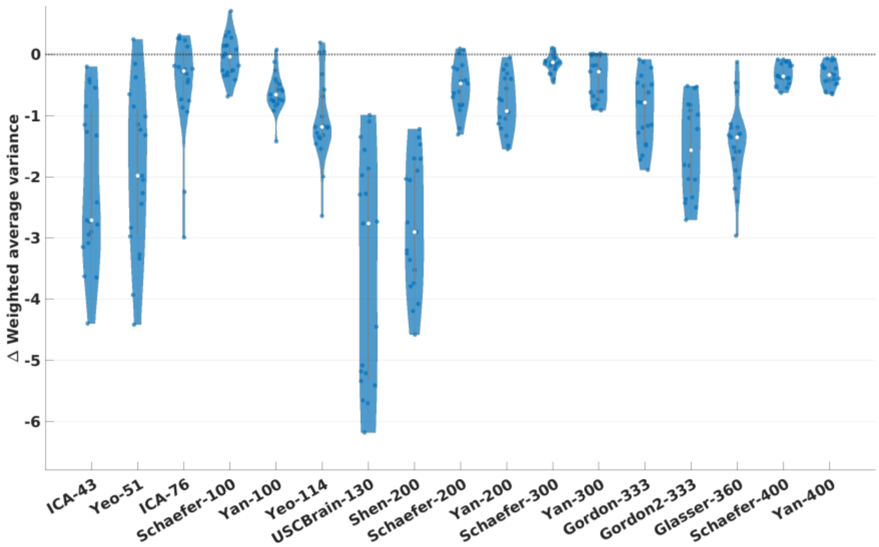

Fig. S2. Weighted average task contrast variance evaluated on 100 HCP test subjects. Each violin plot represents the difference between the weighted average variance for each group-average contrast map with Untamed and the matched baseline (Untamed-baseline). A negative value indicates a lower average within-parcel variance in the Untamed atlas relative to the baseline atlas and vice versa. See text for discussion of statistical significance.

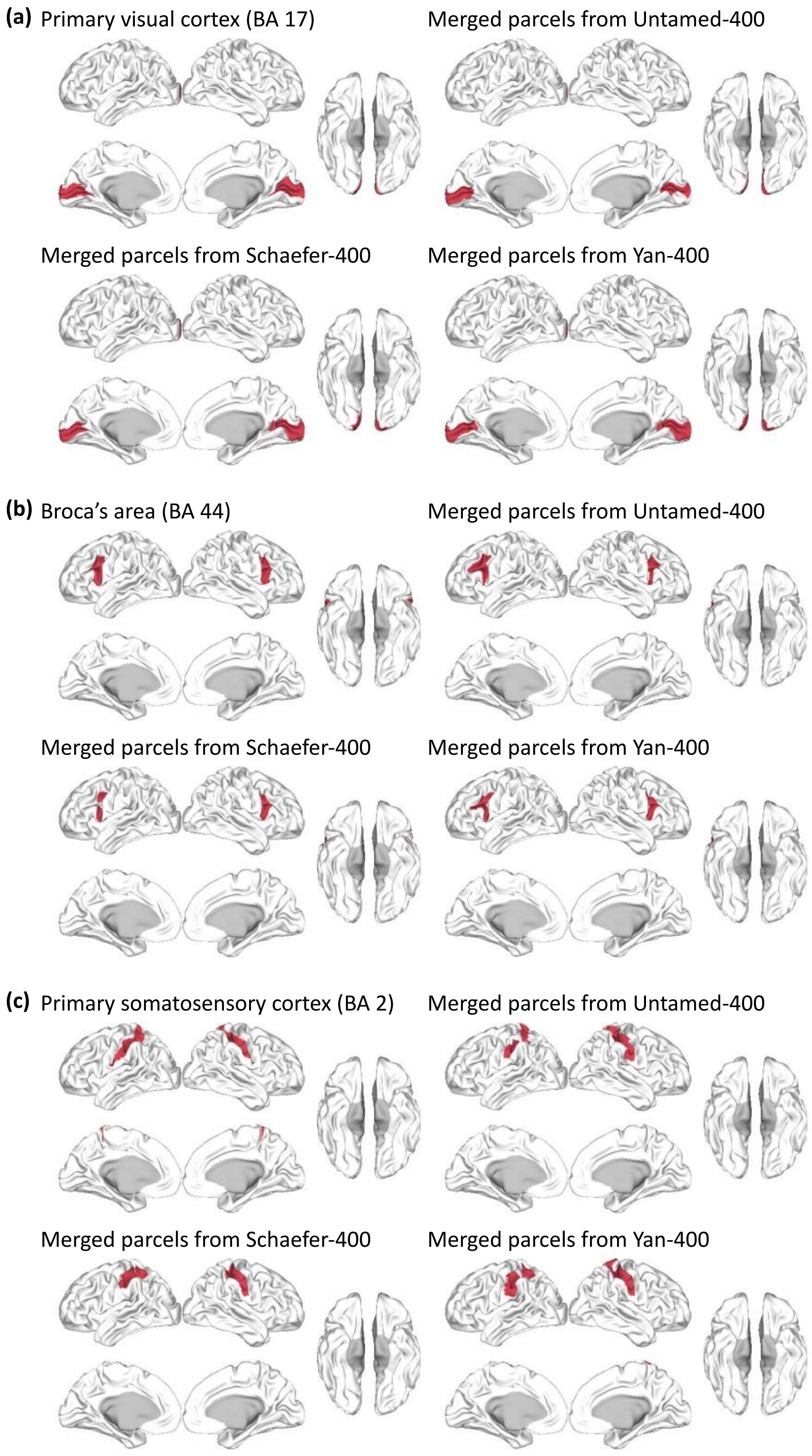

Fig. S3 **(a)** Comparison of merged parcels around Primary visual cortex (BA 17). Dice coefficient of merged parcels with the ROI are 0.766, 0.740, and 0.760 for Untamed, Schaefer, and Yan respectively; **(b)** Comparison of merged parcels around Broca's area (BA 44). Dice coefficient of merged parcels with the ROI are 0.672, 0.697, and 0.714 for Untamed, Schaefer, and Yan, respectively; **(c)** Comparison of merged parcels around Primary somatosensory cortex (BA 2). Dice coefficient of merged parcels with the ROI are 0.786, 0.718, and 0.772 for Untamed, Schaefer, and Yan respectively.

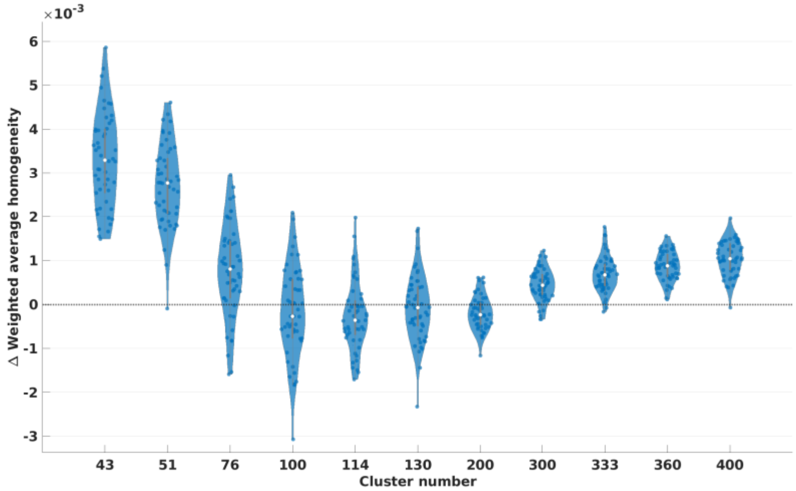

Fig. S4. RSFC weighted average homogeneity across different cluster numbers matching existing parcellations. Each violin plot represents the trial-wise homogeneity difference of average RSFC of training subjects obtained using NetMF-based features for clustering (Untamed method) versus clustering based on the Laplacian eigenvectors (NetMF – Laplacian eigenvectors). A positive value indicates a higher RSFC homogeneity with NetMF than Laplacian eigenvectors.

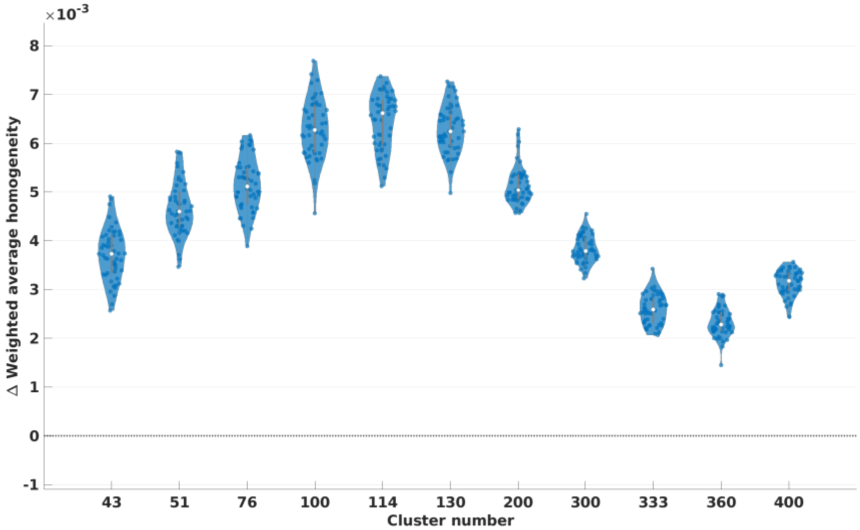

Fig. S5. RSFC average weighted homogeneity comparing NASCAR-based estimation of the graph adjacency matrix (used in Untamed) with computation from Pearson correlation between rsfMRI time-series. Show are the differences between NASCAR-based and Pearson-based adjacencies. A larger value indicates superior homogeneity with NASCAR than Pearson adjacency calculation.

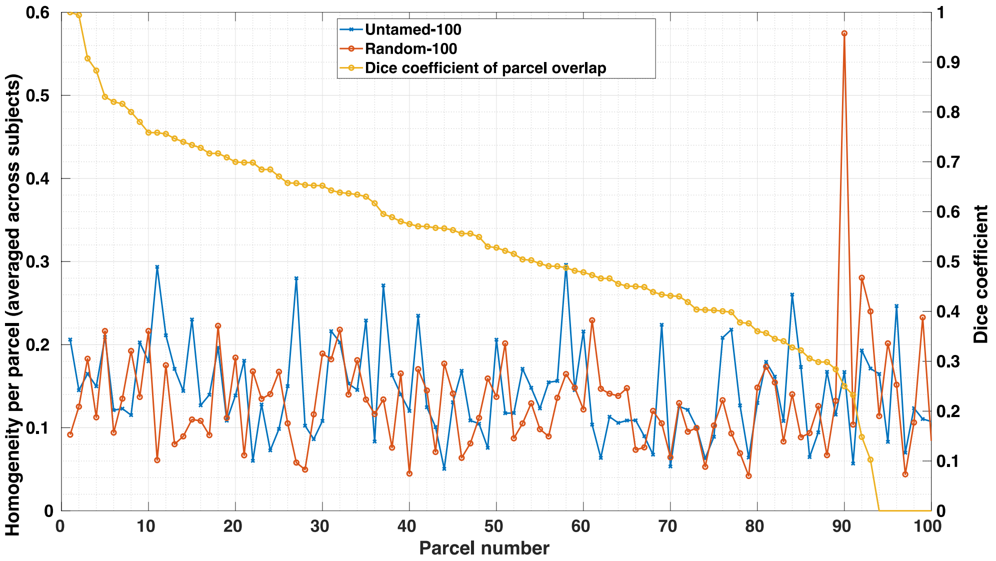

Fig. S6. Parcel-wise RSFC homogeneity scores (averaged across subjects) in Untamed-100 and a Random-100 parcellation computed on Yale rsfMRI test subjects. Homogeneities are sorted by Dice coefficient of matched parcels (also shown) in the two atlases after applying the Hungarian matching algorithm.

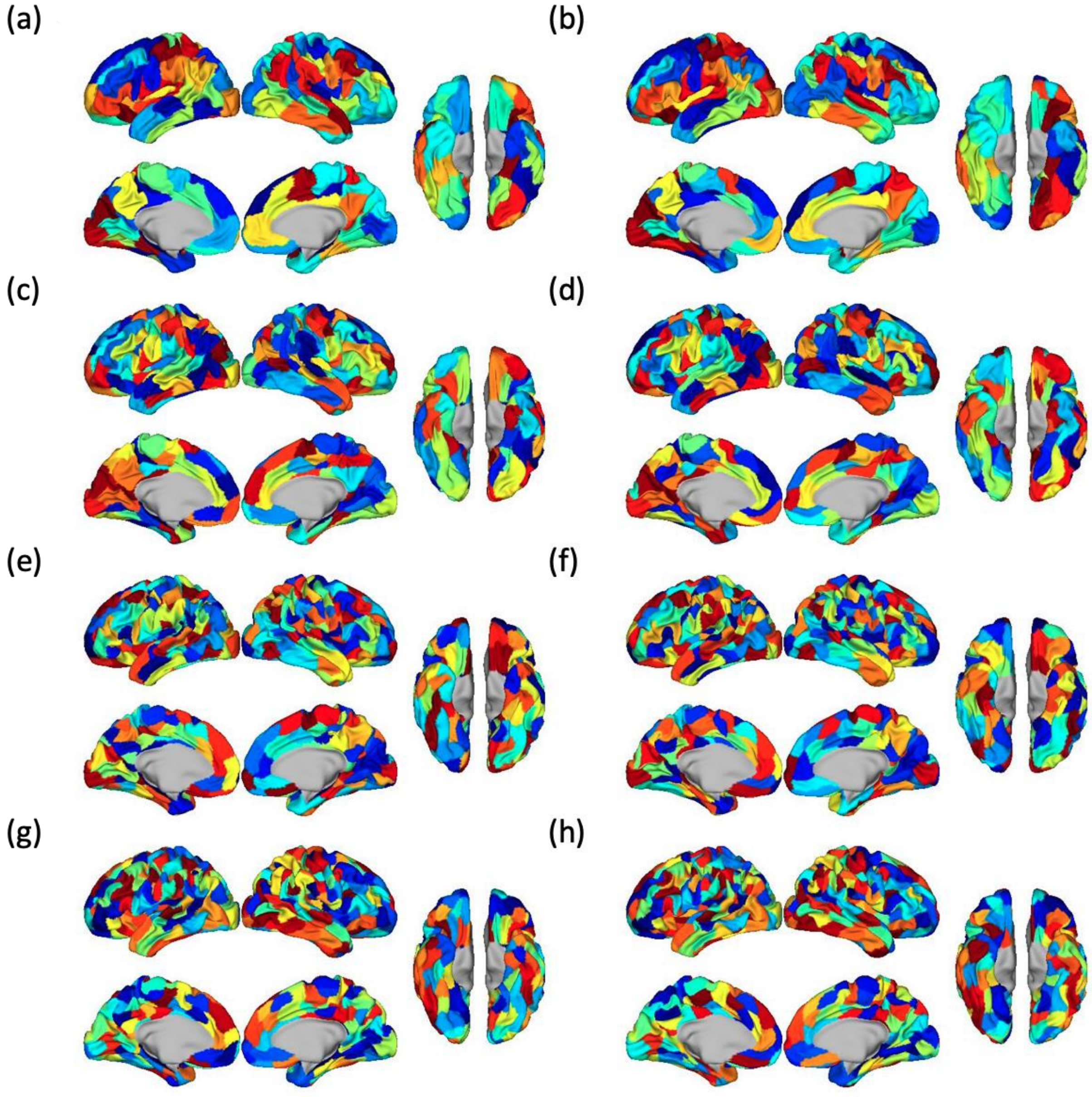

Fig. S7. Parcellation comparison using Schaefer's method: (a)(c)(e)(g): Schaefer-100/200/300/400 and Untamed: (b)(d)(f)(h): Untamed-100/200/300/400. From top to bottom we show 100, 200, 300, and 400 parcels. At each resolution, Hungarian matching was performed to match between the two parcellations to find maximum correspondence in terms of vertex-wise label assignment.
